# Genome‐wide DNA methylation profiles in the raphe nuclei of patients with autism spectrum disorder

**DOI:** 10.1111/pcn.13830

**Published:** 2025-04-24

**Authors:** Keiko Iwata, Kazuhiko Nakabayashi, Keisuke Ishiwata, Kazuhiko Nakamura, Yosuke Kameno, Kenichiro Hata, Hideo Matsuzaki

**Affiliations:** ^1^ Division of Development of Mental Functions, Research Center for Child Mental Development University of Fukui Fukui Japan; ^2^ Department of Functional Brain Activities, United Graduate School of Child Development Hamamatsu University School of Medicine, Osaka University, Kanazawa University, Chiba University, and University of Fukui Osaka Japan; ^3^ Laboratory of Pharmacology, School of Pharmaceutical Sciences Wakayama Medical University Wakayama Japan; ^4^ Department of Maternal–Fetal Biology National Center for Child Health and Development Tokyo Japan; ^5^ Department of Neuropsychiatry Hirosaki University School of Medicine Hirosaki Japan; ^6^ Department of Psychiatry Hamamatsu University School of Medicine Hamamatsu Japan; ^7^ Department of Human Molecular Genetics Gunma University Graduate School of Medicine Maebashi Japan; ^8^ Life Science Innovation Center University of Fukui Fukui Japan

**Keywords:** autism spectrum disorder, epigenetics, serotonergic system

## Abstract

**Aim:**

Autism spectrum disorder (ASD) has a strong genetic basis, yet its genetic complexities remain elusive. Current research highlights environmental factors and epigenetic processes, such as DNA methylation, as crucial in ASD development. This exploratory study addresses a gap in understanding epigenetic regulation in the dorsal raphe (DR)—a region regulating multiple neurotransmitters and implicated in ASD—by examining DNA methylation profiles in postmortem ASD and control brains.

**Methods:**

We comprehensively analyzed genome‐wide DNA methylation profiles in the DR brain region (seven controls and five ASD) using the Infinium HumanMethylation450 BeadChip (Illumina). Additionally, quantitative polymerase chain reaction was used to measure messenger RNA levels of differentially methylated genes in ASD (11 controls and six ASD).

**Results:**

We identified differentially methylated regions (DMRs) between ASD and controls. These DMRs were located among various genomic regions, including promoters, gene bodies, and intergenic regions. Notably, we found hypermethylation in genes related to olfaction (e.g. *OR2C3*), which is regulated by serotonin. Additionally, we observed that the hypomethylation of promoter‐associated CpG islands in *RABGGTB*, a gene related to autophagy and synaptic function, corresponded with its increased expression.

**Conclusions:**

Our findings reveal extensive DNA methylation changes in critical genomic regions, shedding light on potential mechanisms underlying ASD. The identification of *RABGGTB* as a novel candidate gene, not listed in the SFARI database, underscores its significance and warrants further research to explore its role in ASD diagnosis. This study enhances our understanding of the epigenetic landscape in ASD, emphasizing the interplay between genetic and environmental factors in its pathophysiology.

Autism spectrum disorder (ASD) is a neurodevelopmental disorder characterized by severe and sustained impairment of social interaction, deviance in communication, and patterns of behavior and interest that are restricted or stereotyped. The global prevalence of ASD is estimated to be approximately 0.1%,^1^ although it varies significantly between countries. Although twin studies have provided evidence for a strong genetic component for the condition, and many candidate genes have been reported, the genetic mechanisms underlying ASD remain unclear. It has been reported that genetic heritability is lower than previously estimated and that environmental factors have a greater impact on the development of ASD.^2^ Several reports have suggested that exposure to environmental factors during the earliest days of fetal life, such as chemicals, stress, and viral infections, may contribute to ASD.^3^, ^4^, ^5^

The epigenetic processes that modulate gene expression, such as DNA methylation and histone modification, are considered to be at the interface between genetic and environmental factors. Parental imprinting of a chromosome (such as Angelman syndrome and Prader–Willi syndrome) and X chromosome inactivation (such as Fragile X syndrome and Rett syndrome) are two well‐characterized epigenetic processes.^6^, ^7^, ^8^ These syndromes share neurological and behavioral symptoms with ASD.

Several investigators have reported differentially methylated regions (DMRs) in the postmortem brains of individuals diagnosed with ASD. DNA methylation in the brain exhibits region‐specific characteristics, with significant variability reflecting the functional and cellular diversity of brain regions.^9^ However, most studies on ASD focus on the cortex and cerebellum. Early research highlighted methylation abnormalities in ASD‐related genes, such as *UBE3A*, *MECP2*, *OXTR*, and *SHANK3*, in the temporal, frontal, cerebral cortices, and cerebellum, respectively.^10^, ^11^, ^12^, ^13^ Contrastingly, no differentially methylated CpG sites were found for *MECP2*, *UBE3A*, *BCL2*, *RORA*, or *OXTR* in the occipital or cerebellar cortex in one study.^14^ Thereafter, using a bump‐hunting approach, differentially methylated genes such as *PRRT1*, *C11orf21*, *ZFP57*, and *SDHAP3* were identified in the temporal cortex and cerebellum.^15^ Hypomethylated genes related to synaptic pruning and microglial function, including *C1Q*, *C3*, *ITGB2 (C3R)*, and *TNF‐α*, were also reported in these regions.^16^ Additionally, genome‐wide DNA methylation studies in the prefrontal cortex of individuals with ASD suggested microglial–neuron interactions as potential contributors to pathology.^17^

A growing body of evidence identifies the dorsal raphe (DR), located in the ventral central gray matter of the mesencephalon and rostral pons, as a key region involved in ASD. The DR contains the largest population of serotonergic neurons, which project extensively to the forebrain and peripheral regions, playing a central role in serotonergic regulation and ASD‐related mechanisms.^18^, ^19^ Notably, hyperserotonemia—elevated serotonin levels in blood and platelets—has been consistently reported in individuals with ASD.^20^, ^21^, ^22^, ^23^, ^24^ Additionally, reduced membrane expression and serotonin transport capacity of the serotonin transporter have been observed in patients with ASD.^25^, ^26^ On the other hand, the DR nucleus comprises various cell types beyond serotonergic neurons, further diversifying its functional role.^27^ In animal models, optogenetic activation of the DR has been shown to improve social deficits in ASD models, including Shank3 knockout mice and 16p11.2 knockout mice.^28^, ^29^ Moreover, mutations in Taok1, a gene preferentially expressed in the DR, have been associated with ASD‐like phenotypes, highlighting the importance of the DR in ASD pathophysiology.^30^ Environmental factors, such as peripheral immune activation and exposure to stress hormones, have been shown to alter neuronal activity in the DR, further linking this region to ASD mechanisms.^31^, ^32^ Despite these insights, DNA methylation profiles in the DR in the context of ASD remain unexplored, representing a critical gap in understanding the epigenetic regulation of this brain region.

Here, we conducted an exploratory investigation of genome‐wide DNA methylation profiles in the DR region from postmortem brain tissues of individuals with ASD and individuals not diagnosed with ASD (controls). This study aims to generate preliminary insights that can inform future hypothesis‐driven research. Given the recognized importance of methylation nonpromoter regions in addition to promoter regions,^33^, ^34^, ^35^ we investigated the methylation status using the Infinium HumanMethylation450 BeadChip (Illumina), which can measure the DNA methylation levels of CpG dinucleotides in the promoter regions, including 5′ untranslated region (UTR), as well as in the intragenic (corresponding gene bodies), intergenic, and 3′ UTR.^36^, ^37^

## Methods

### Postmortem brain tissues

The ethics committee of the Hamamatsu University School of Medicine approved this study. Postmortem brain tissues were obtained through the Autism Tissue Program (ATP), which coordinated the distribution of ASD brain samples for research purposes at the time of sample acquisition. Following formal application and approval by ATP's review process, brain samples were provided by the University of Maryland Brain and Tissue Bank (UMBTB) and the Harvard Brain Tissue Resource Center (HBTRC). These institutions collected and preserved postmortem brain samples under standardized protocols to ensure sample integrity and research feasibility. Frozen postmortem brain tissues from the DR regions of five individuals with ASD and seven controls were analyzed. ASD was diagnosed according to the DSM‐IV‐TR, and the diagnosis was confirmed by the Autism Diagnostic Interview—revised (ADI‐R). Information obtained from the next of kin showed that none of the controls had any known history of neuropsychiatric disorders or illicit drug use. The demographic characteristics of the samples are described in Table S1. The Mann–Whitney *U* test was used to evaluate the differences in age and postmortem interval (PMI) between the ASD and control groups. Fisher exact test was used to evaluate the differences in sex and ethnicity between the ASD and control groups.

Significance was set at *P* < 0.05. All statistical analyses were performed using statistical analysis software (SPSS version 12.0J, IBM).

### Genome‐wide DNA methylation array

Genomic DNA was extracted from the brain tissues of male patients (Table S1, C1‐C7 and A1‐A5) using an AllPrep DNA/RNA Mini Kit (Qiagen). The DNA samples were treated with bisulfite using the EZ DNA Methylation Kit (Zymo Research). Genome‐wide DNA methylation was assessed using the Infinium HumanMethylation450 BeadChip Kit (Illumina), which covers >450K CpG sites and targets 96% of CpG islands (CGIs) in the human genome.^36^ Illumina GenomeStudio software (Illumina) was used to extract signal intensities for each probe and perform initial quality‐control checks. To ensure stringent data quality, probes with a detection *P* > 0.05 or a blank *β*‐value in any sample were removed among all individuals. Because SNPs may affect methylation in nearby CpG dinucleotides,^38^, ^39^, ^40^ we excluded probes associated with SNPs (Okamura *et al*. Genomics Data: http://www.sciencedirect.com/science/article/pii/S2213596015300933).

### Methylation array data analysis

The subtract background function and the controls normalization function of GenomeStudio were applied. The relative methylation level of each CpG site was calculated as the ratio of the normalized signal from the methylated probe to the sum of the normalized signals of the methylated and nonmethylated probes. DNA methylation levels were quantified using *β*‐values, which range from 0 (completely unmethylated) to 1 (fully methylated) and represent the proportion of methylation at each CpG site. To compare methylation differences between the ASD and control groups, Δ*β*‐values were calculated as the difference in mean *β*‐values between groups (Δ*β* = β_ASD–β_control). For group analysis, a filtering criterion (Δ*β* = average *β*‐value of ASD–average *β*‐value of control) > 0.1 or < −0.1 was applied, and *P* < 0.05 according to the Mann–Whitney *U* test was used to assign significant differentially methylated sites. We did not use the Bonferroni method to adjust the statistical significance to the number of tests performed.^41^ The effect size *r* for the Mann–Whitney *U* test was calculated to evaluate the magnitude of group differences. The formula used was: $r=\frac{Z}{\sqrt{N}}$ where *Z* is the standardized test statistic from the Mann–Whitney *U* test, and *N* is the total sample size. *Z*‐scores were obtained using statistical software. Effect size *r* was interpreted as small (0.1 ≤ *r* < 0.3), medium (0.3 ≤ *r* < 0.5), or large (*r* ≥ 0.5). Categorical variables were analyzed using Fisher exact test. All statistical analyses were performed using SPSS version 12.0J (IBM). The data discussed in this publication have been deposited in the Gene Expression Omnibus (GEO; http://www.ncbi.nlm.nih.gov/geo/) of NCBI and are accessible through the GEO Series accession number GSE242427.

### EM‐amplicon sequencing

Genomic DNA (50 ng) was fragmented using an S220 focused ultrasonicator (Covaris). The sheared DNA was then treated with the NEBNext Enzymatic Methyl‐Seq Conversion Module (New England Biolabs, E7120) to enzymatically convert the unmethylated cytosine to uracil while leaving the 5‐methylcytosine unaltered. Polymerase chain reaction (PCR) amplification was performed in a total volume of 20 μL, which contained 10 ng of enzymatically converted DNA, 20 pmol of each primer, dNTPs (2.5 mM each), 1.5 U of ExTaq HS (Takara), and the supplied buffer. The sequences of the PCR primers used are 5′‐TTTTTAAGGTTAATGATGTGTTGGTAT‐3′ and 5′‐CAACTAAATTTAAATACTACAAAAAAAA‐3′, which were designed to amplify a 211 bp fragment corresponding to chr1: 76,253,567‐76,253,777 (hg19). The PCR products were purified using AMPure XP beads (Beckman Coulter). The purified PCR products (1 ng) were ligated with sequencing adaptors using the NEBNext Ultra II DNA Library Prep Kit for Illumina (New England Biolabs, E7645), which were amplified by six cycles of PCR and purified using Sample Purification Beads. The final products were sequenced using the MiSeq Reagent Nano Kit v2 (Illumina, MS‐103‐1001) on a MiSeq platform (Illumina). Fastq files were trimmed using trim_galore (v. 0.6.6) (https://www.bioinformatics.babraham.ac.uk/projects/trim_galore/) and aligned using Bismark (v. 0.22.3) (https://www.bioinformatics.babraham.ac.uk/projects/bismark/). The resultant bam files were subjected to methylKit^42^ to determine the methylation ratio at cg08702915 (chr1: 76,253,688 (hg19)). Mann‐Whitney *U* test (Wilcoxon signed rank test) was performed using the wilcox.test function of R (version 4.4.2) to assess the statistical significance of DNA methylation difference between ASD (A1–A5) and control (C1–C7) samples.

### Quantitative PCR

Messenger RNA (mRNA) was extracted from the brain tissues from both male and female patients (Table S1, C1–C11 and A1–A6) using an AllPrep DNA/RNA Mini Kit (Qiagen). First‐strand complementary DNA (cDNA) was synthesized from the extracted RNA samples using the SuperScript III First‐Strand Synthesis System (Invitrogen). cDNA was used for quantitative real‐time PCR, which was performed using the SYBR Green Master Mix (Bio‐Rad). The relative quantification of the *OR2C3*, *COLEC11*, *DCAKD*, *RABGGTB*, and *ZBTB40* expression levels was performed using the ΔΔC_T_ method, with the expression of the *ACTB* gene used as the internal control. Primer sequences are available upon request. The Mann–Whitney *U* test was used to evaluate the gene expression levels. Evaluation of the relationships between methylations and gene expressions was performed using Spearman rank correlation coefficient. *P* values <0.05 were considered statistically significant (SPSS version 28.0J, IBM.).

## Results

### Characteristics of the participants and brain samples

We used only male patients for methylation analysis because the DNA methylation levels at several autosomal loci in the brain are sex‐dependent.^43^, ^44^ The demographic characteristics of participants (five patients with ASD and seven controls) are described in Tables S1. There were no significant differences in age, ethnicity, or PMI between the ASD and control groups (Table S2).

### Brain genome‐wide methylation analysis: average of the controls versus average of the patients with ASD

We used a DNA methylation microarray that assays the DNA methylation status of >450,000 CpG sites, including promoters, as well as the corresponding gene bodies and 3′ UTR, in addition to intergenic regions derived from GWAS studies.^36^, ^37^ This microarray platform produces DNA methylation data with an accuracy similar to that of other approaches, such as methylated DNA immunoprecipitation sequencing, methylated DNA capture by affinity purification, and reduced‐representation bisulfite sequencing.^45^

**Fig. 1:**
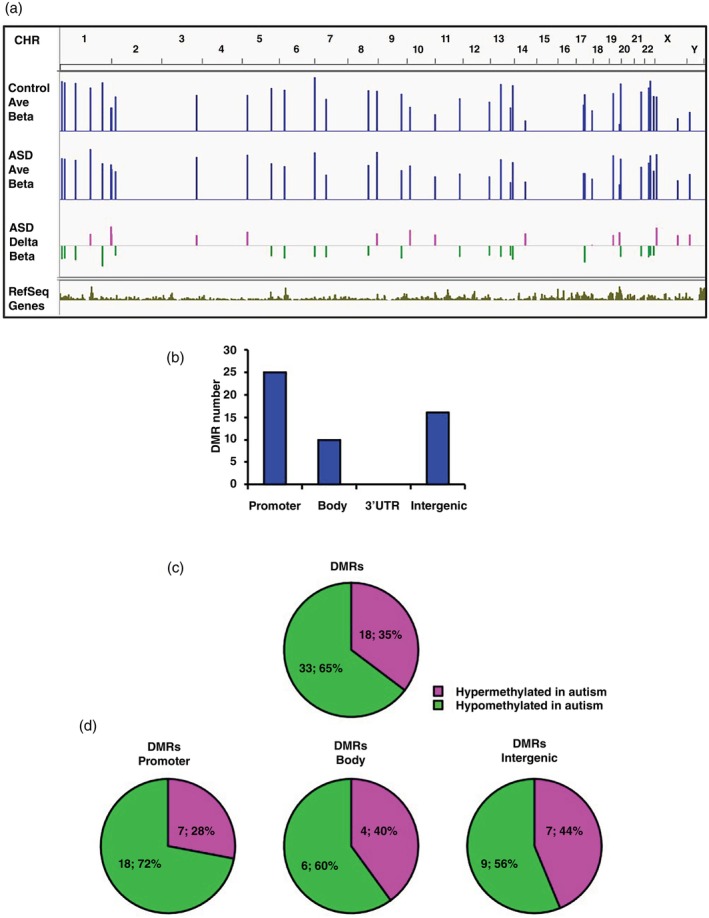
Brain genome–wide methylation analysis: average of controls versus average of patients with autism spectrum disorder (ASD). (a) Integrative genomics viewer–based view of sequenced differentially methylated region (DMR) tracks of differences in average methylation values between the controls and patients with ASD. Average methylation values that were significantly (*P* < 0.05) increased or decreased in patients with ASD compared with the controls are shown (blue bars). The average methylation values are expressed as *β*‐values (0–1). Delta *β*‐values are colored in pink (increased) or green (decreased). (b) DMR distribution among different genomic sequences. (c) Distribution of DMRs according to the direction of the DNA methylation change (d) and among different genomic sequences. CHR, chromosome; UTR, untranslated region.

We identified 51 DMRs between the ASD and control groups, all of which had effect sizes of *r* > 0.5, indicating a large effect (Table 1). The DMRs were distributed among almost all human chromosomes (Fig. 1a). As shown in Fig. 1b, DMRs were identified in the promoter (25 CpG sites), body (10 CpG sites), and intergenic (16 CpG sites) regions, and none of them were identified in 3′ UTR. In individuals with ASD, 35% (18 sites) of the DMRs were hypermethylated and 65% (33 sites) were hypomethylated (Fig. 1c). Further analysis by regions, 28% of the DMRs in the promoter region (seven sites), 40% in the gene body region (four sites), and 44% in intergenic region (seven sites) corresponded to sequences with increased methylation, whereas 72% of the DMRs in the promoter region (18 sites), 60% in the gene body region (six sites), and 56% in the intergenic regions (nine sites) represented sequences with increased hypomethylation (Fig. 1d). There were no significant differences in the ratios of hypermethylation to hypomethylation between these three regions (promoter vs body, *P* = 0.689; promoter vs intergenic, *P* = 0.332; body vs intergenic, *P* = 1.000; Fisher exact test).

**Table 1:** Hypermethylated and hypomethylated DMRs in the patients with ASD

Illumina target ID	CpG map site	Gene symbol	Protein name	Chromosome	FGD	CpG context	Delta beta	*P‐*value	Effect size (*r*)
cg08579577	8,751,402			X	Intergenic	Island	0.199,306	0.030	0.63
cg12754571	247,694,271	*OR2C3*	Olfactory receptor 2C3	1	Promoter	Island	0.190,023	0.048	0.59
cg12758973	247,694,275	*OR2C3*	Olfactory receptor 2C3	1	Promoter	Island	0.177,698	0.048	0.59
cg13666323	8,751,190			X	Intergenic	N_Shore	0.182,397,8	0.030	0.63
cg15690985	15,016,559			10	Intergenic		0.152,441,2	0.048	0.59
cg10521948	8,751,524			X	Intergenic	Island	0.136,865,8	0.030	0.63
cg22863700	28,928,346			5	Intergenic	Island	0.133,109	0.010	0.73
cg15690347	50,931,515	*SPIB*	Spi‐B transcription factor (Spi‐1/PU.1 related)	19	Body	Island	0.129,354	0.018	0.68
cg09974661	3,642,634	*COLEC11*	Collectin‐11	2	Promoter	Island	0.119,703	0.005	0.77
cg25092838	57,279,115	*OTX2OS1*		14	Promoter	Island	0.116,478	0.048	0.59
cg09374673	144,986,488			8	Intergenic	N_Shelf	0.115,051,7	0.048	0.59
cg02090762	79,503,859	*FSCN2*	Fascin‐2	17	Body	Island	0.111,713	0.003	0.82
cg15448220	150,897,856	*SETDB1*	Histone‐lysine N‐methyltransferase SETDB1	1	Promoter	N_Shore	0.109,267	0.018	0.68
cg21191514	2,187,921	*TH*	Tyrosine 3‐monooxygenase	11	Body	Island	0.107,257	0.005	0.77
cg10363397	15,864,778			Y	Intergenic	Island	0.103,794,2	0.018	0.68
cg17872886	3,642,732	*COLEC11*	Collectin‐11	2	Promoter	Island	0.103,625	0.030	0.63
cg10503326	170,028,283			3	Intergenic		0.103,429,4	0.003	0.82
cg08840441	19,748,461	*GMIP*	GEM‐interacting protein	19	Body	Island	0.103,102	0.010	0.73
cg01813224	113,815,930			X	Intergenic	N_Shore	0.101,324,2	0.030	0.63
cg10600889	37,123,767	*FBXO47*	F‐box protein 47	17	Promoter	Others/Open sea	0.102,159	0.048	0.59

Illumina target ID	CpG map site	Gene symbol	Protein name	Chromosome	FDG	CpG context	Delta beta	*P*‐value	Effect size (*r*)
cg17178900	205,818,956	*PM20D1*	Peptidase M20 domain containing 1	1	Body	Island	−0.201,974	0.048	0.59
cg25538415	43,129,957	*DCAKD*	Dephospho‐CoA kinase domain‐containing protein	17	Promoter	S_Shore	−0.165,945	0.048	0.59
cg08702915	76,253,688	*RABGGTB*	Geranylgeranyl transferase type‐2 subunit beta	1	Promoter	Others/Open sea	−0.143,597	0.030	0.63
cg21415347	22,777,839	*ZBTB40*	Zinc finger and BTB domain containing 40	1	Promoter	N_Shore	−0.141,511	0.030	0.63
cg09266979	22,777,744	*ZBTB40*	Zinc finger and BTB domain containing 40	1	Promoter	N_Shore	−0.113,397	0.018	0.68
cg09966204	109,741,117	*MYO16*	Unconventional myosin‐XVI	13	Body	Others/Open sea	−0.138,849	0.030	0.63
cg27193005	26,382,695	*BTN2A2*	Butyrophilin subfamily 2 member A2	6	Promoter	Others/Open sea	−0.136,152	0.048	0.59
cg24347070	26,382,817	*BTN2A2*	Butyrophilin subfamily 2 member A2	6	Promoter	Others/Open sea	−0.121,953	0.030	0.63
cg20978923	12,184,574	*TNFRSF8*	Tumor necrosis factor receptor superfamily, member 8	1	Promoter	Others/Open sea	−0.134,664	0.003	0.82
cg14437002	115,841,105			9	Intergenic	Island	−0.130,512,1	0.030	0.63
cg10895875	56,242,407			7	Intergenic	Island	−0.127,487,1	0.030	0.63
cg07496173	57,472,222			7	Intergenic	N_Shore	−0.127,325,7	0.030	0.63
cg20115597	56,516,248	*LOC650226*	Ankyrin repeat domain 26 pseudogene	7	Promoter	S_Shore	−0.121,551	0.048	0.59
cg26262573	56,516,129	*LOC650226*	Ankyrin repeat domain 26 pseudogene	7	Promoter	S_Shore	−0.110,134	0.030	0.63
cg17328716	56,516,243	*LOC650226*	Ankyrin repeat domain 26 pseudogene	7	Promoter	S_Shore	−0.100,433	0.048	0.59
cg17623879	19,436,004	*C22orf39*	Chromosome 22 open reading frame 39	22	Promoter	S_Shore	−0.115,134	0.003	0.82
cg23316599	57,271,379			7	Intergenic	Island	−0.112,368,5	0.030	0.63
cg17949813	1,801,521			7	Intergenic		−0.111,232,1	0.005	0.77
cg20905796	122,376,861			11	Intergenic		−0.110,877,4	0.018	0.68
cg25802888	57,742,112	*AURKC*	Aurora kinase C	19	Promoter	Island	−0.110,374	0.030	0.63
cg16096766	52,419,714	*FLJ37307*		13	Promoter	Others/Open sea	−0.109,718	0.030	0.63
cg18593945	32,015,231	*KRTAP20‐3*	Keratin‐associated protein 20–3	21	Promoter	Others/Open sea	−0.106,590	0.048	0.59
cg19889580	143,584,273	*KCTD16*	BTB/POZ domain‐containing protein KCTD16	5	Promoter	N_Shore	−0.106,350	0.048	0.59
cg02946570	21,023,387	*C2orf43*	UPF0554 protein C2orf43	2	Promoter	S_Shore	−0.102,398	0.010	0.73
cg19200953	100,470,738	*CLYBL*	Citrate lyase subunit beta‐like protein, mitochondrial	13	Body	Others/Open sea	−0.102,201	0.010	0.73
cg09637757	104,831,516	*RIMS2*	Regulating synaptic membrane exocytosis protein 2	8	Promoter	Others/Open sea	−0.101,707	0.003	0.82
cg03454920	49,826,270			22	Intergenic		−0.101,658,2	0.003	0.82
cg17734698	129,127,968	*TMEM132C*	Transmembrane protein 132C	12	Body	Others/Open sea	−0.101,009	0.048	0.59
cg13797031	29,977,742	*NIPSNAP1*	Protein NipSnap homolog 1	22	Promoter	S_Shore	−0.100,595	0.030	0.63
cg21096718	35,577,353	*ACACA*	Acetyl‐CoA carboxylase 1	17	Body	Others/Open sea	−0.100,410	0.010	0.73
cg18605975	78,774,723	*RPTOR*	Regulatory‐associated protein of MTOR, complex 1	17	Body	N_Shore	−0.100,024	0.003	0.82

Functional genomic distribution (FGD) is classified into different groups: promotor, body, and intergenic. The 2 kb regions flanking the CpG island (CGI) are defined as CGI shores: the upstream 2 kb as the north shore (N_Shore) and the downstream 2 kb as the south shore (S_Shore). The 4 kb regions upstream and downstream of the CGI are defined as CGI shelves: the north shelf (N_Shelf). Delta *β*‐values are colored in pink (indicating an increase) or green (indicating a decrease). ASD, autism spectrum disorder; DMR, differentially methylated region.

Next, we focused on the DMRs identified in the genic regions (35 CpG sites), including the promoter and body (Table 1). We detected 11 significantly hypermethylated CpG sites in 10 genes, such as *OR2C3* and *SPIB*, in patients with ASD compared with the controls, as well as 24 hypomethylated CpG sites in 20 genes, such as *PM20D1* and *DCAKD*, in patients with ASD compared with the controls (Table 1). These genes represent novel candidate ASD genes. In the intergenic region, we found a notable hypermethylated DMR region on the X chromosome, located just 5′ upstream of *HTR2C*, which encodes the serotonin receptor 2C (Fig. S1).

### mRNA expression of differentially methylated genes in ASD

**Fig. 2:**
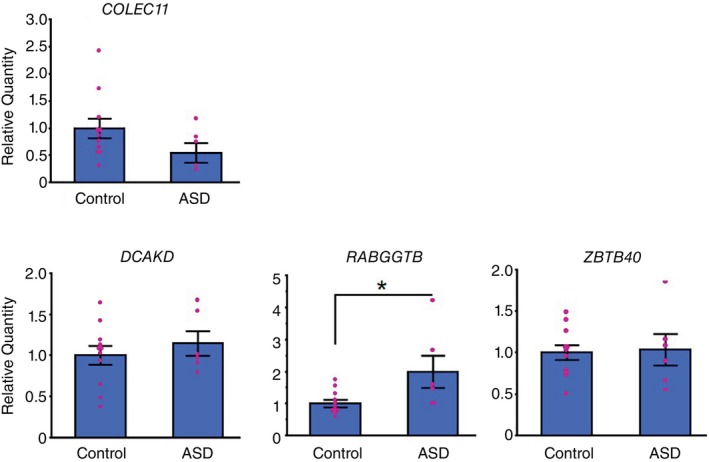
Expression of the *COLEC11*, *DCAKD*, *RABGGTB*, and *ZBTB40* genes in the raphe region of postmortem brains. Comparison of the gene levels in the raphe region of postmortem brains from controls and patients with autism spectrum disorder (ASD). Data are presented as mean ± SEM. *n* = 11 controls and *n* = 6 patients with ASD. *P < 0.05 versus control using the Mann–Whitney *U* test.

Next, we focused on the mRNA expression levels of genes that demonstrated differential methylation in ASD. The study involved six patients with ASD and 11 controls including female samples, and their demographic characteristics are outlined in Table S1. There were no significant differences between the two groups in terms of age, sex, ethnicity, or PMI (Table S3). From the top four hypermethylated and hypomethylated regions with the largest methylation abnormalities (excluding intergenic regions), we selected *OR2C3*, *COLEC11*, *DCAKD*, *RABGGTB*, and *ZBTB40* as genes with abnormally methylated CGIs in their promoter regions. Subsequently, we analyzed the expression levels of these genes (Table 1). Our findings indicate a significant increase in the mRNA expression level of the *RABGGTB* gene in individuals with ASD compared with the control group (*P* = 0.027; Fig. 2), which supports a significant decrease in methylation of the gene. Furthermore, we confirmed a significant decrease in methylation at cg08702915 of *RABGGTB*; this DMR was identified by the genome‐wide DNA methylation array (Table 1) through EM‐amplicon sequencing (*P* = 0.030; Table 2 and Fig. S2). However, *OR2C3* was below the detection sensitivity threshold, and no significant differences in gene expressions were observed between the two groups for the *COLEC11*, *DCAKD*, and *ZBTB40* genes (Fig. 2). Additionally, we conducted an analysis using only male samples, which were included in the methylation array analysis. First, the gene expression analysis revealed a tendency similar to that observed in Fig. 2, indicating that the mRNA expression level of the *RABGGTB* gene tends to increase (*P* = 0.073; Fig. S3). Furthermore, we examined the correlation between *COLEC11*, *DCAKD*, *RABGGTB*, and *ZBTB40* gene expression and their methylation (AVG_Beta values) obtained from the methylation array for each site. Among the genes analyzed, only the *RABGGTB* gene exhibited a negative relationship trend between its expression and methylation levels (*P* = 0.071; Fig. S4).

**Table 2:** DNA methylation rates at cg08702915 (chr1: 76,253,688 (hg19)) located in intron 2 of *RABGGTB*

Sample	Chromosome	Pos (hg19)	Meth (%)	Meth (%)_average	*t*‐Test
					*P*‐value
A1‐bs‐cg08702915	1	76,253,688	38.9		
A2‐bs‐cg08702915	1	76,253,688	37.4		
A3‐bs‐cg08702915	1	76,253,688	43.8	46.6	
A4‐bs‐cg08702915	1	76,253,688	69.4		
A5‐bs‐cg08702915	1	76,253,688	43.6		
C1‐bs‐cg08702915	1	76,253,688	57.1		
C2‐bs‐cg08702915	1	76,253,688	80.3		0.030
C3‐bs‐cg08702915	1	76,253,688	82.4		
C4‐bs‐cg08702915	1	76,253,688	74.5	66.5	
C5‐bs‐cg08702915	1	76,253,688	44.0		
C6‐bs‐cg08702915	1	76,253,688	62.9		
C7‐bs‐cg08702915	1	76,253,688	64.3		
				−19.9	

## Discussion

The current study represents an exploratory investigation into the DNA methylation profiles of the DR in ASD. Given the relatively small sample size and the complexity of the data, the findings should be interpreted with caution. This research provides a foundation for future studies that can validate these results in larger and more diverse cohorts.

The study revealed the presence of significant differences in DNA methylation profiles in the DR region of postmortem brains between patients with ASD and controls. Furthermore, DMRs were detected not only in promoter regions but also in gene bodies and intergenic regions.

Among the DMRs detected in this study, we identified several located in genes associated with the serotonergic system, such as *OR2C3*, which encodes the olfactory receptor 2C3. This gene exhibited hypermethylation at two promoter CGIs; however, the gene expression of *OR2C3* was below the detection sensitivity threshold in quantitative PCR, preventing us from determining whether there were any changes in gene expression in ASD. The olfactory bulb is one of the most densely innervated targets of serotonin fibers from the raphe brain region.^18^ In *Drosophila*, serotonin was shown to excite both projection and local neurons and to increase the presynaptic inhibition of olfactory receptor neurons. Moreover, it has been proposed to suppress weak olfactory receptor neuron responses.^46^ Intriguingly, olfactory impairment and an atypical sensory response have been identified in individuals with ASD,^47^ as well as in other individuals diagnosed with a developmental disorder.^48^, ^49^ Furthermore, olfactory dysfunction has been linked to the clinical phenotype of ASD.^50^ Given these associations, the hypermethylation of the promoter region of the olfactory receptor may contribute to olfactory processing alterations observed in ASD, although further investigation is needed to clarify this relationship.

We also observed that the serotonin‐related gene *HTR2C* was located in the flanking region of the DMR detected in the intergenic region. *HTR2C*, which is located on the X chromosome, encodes the serotonin receptor 2C. The serotonin receptor 2C is suggested to be involved in social behavior.^51^ In mice, the expression of *Htr2c* is epigenetically regulated by the binding of the methyl‐CpG binding protein 1 (Mbd1) to its promoter.^52^ The loss of Mbd1 has been shown to lead to the overexpression of *Htr2c*.^52^ Moreover, mutant mice lacking Mbd1 display several core deficits associated with ASD.^52^ Research has demonstrated that the methyl‐CpG–binding protein 2 preferentially binds to intragenic and intergenic regions, with less affinity for the methylated promoters of active genes.^35^ However, it is not well understood whether Mbd1 binds intergenic methylated regions as well. It will be necessary to investigate the possibility of Mbd1 binding to intergenic methylated regions.

A multitude of DMRs are discovered within gene bodies. DNA methylation principally occurs at cytosine residues located in CpG dinucleotides.^53^ Although CpG dinucleotides are statistically underrepresented in the genome, they are concentrated at CGIs, which frequently coincide with promoter or gene‐regulatory regions. Previous research indicates that gene body hypermethylation is linked to diminished gene expression, particularly in highly expressed genes.^33^ In the context of human genomes, it has been confirmed that methylated gene body regions correlate with elevated levels of gene transcription.^54^, ^55^ Moreover, gene body methylation impacts processes such as histone modification,^56^ alternative splicing,^57^ and spurious transcription.^58^ Concurrently, numerous studies have revealed the close relationship between abnormal gene body methylation and gene expression, growth, differentiation, and development.

Recently, it has been demonstrated that the intergenic DNA hypomethylation resulting from a dysfunction in the *trans*‐regulatory pathways of the histone methyltransferase NSD1 and the DNA methyltransferase DNMT3A serves as a mechanistic link between two phenotypically overlapping human overgrowth syndromes, both of which also exhibit a developmental delay.^59^ In the current study, the ratios of the hypermethylation to the hypomethylation of DMRs were consistent across promoter, intergenic, and body regions. Recently, Correlated Regions of Systemic Interindividual Variation (CoRSIVs) have been identified as enriched in intergenic regions and are thought to play a significant role in epigenetic regulation, independent of direct gene function.^60^ These regions have also been implicated in various human disorders, including psychiatric conditions.^60^ Given the observed aberrant methylation within intergenic regions in ASD, it will be crucial to investigate how this phenomenon influences gene expression and contributes to the pathophysiological mechanisms of ASD. Further exploration of this subject is warranted for a comprehensive understanding of this disorder.

In this study, we confirmed that the increased expression of *RABGGTB* corresponds to the hypomethylation of the gene's promoter‐associated CGIs. RABGGTB is a *β* subunit of GGTase II, one of the prenyltransferases responsible for protein prenylation.^61^ Prenylation of proteins plays a vital role in their membrane binding and localization. Rab7, a member of the Ras GTPase superfamily, is involved in autophagy, a cellular process essential for maintaining cellular homeostasis by degrading and recycling damaged or redundant cellular components.^62^ The function of Rab7 depends on its localization to the plasma membrane, and RABGGTB performs a critical role in modulating autophagy by mediating the prenylation of Rab7.^62^ The prenylation of Rab3a induced by RABGGTB is essential for regulating vesicle fusion and neurotransmitter release at the nerve terminal.^63^, ^64^ In addition, an increased gene expression level of *RABGGTB* has been reported in the postmortem prefrontal cortex of individuals with schizophrenia compared with healthy controls.^65^ Moreover, decreased protein expression levels have been observed in the dorsolateral prefrontal cortex of individuals with schizophrenia compared with healthy controls.^66^ Accumulated evidence suggests that synaptic dysfunction contributes to the pathogenesis of ASD.^67^ Therefore, considering prenylation's role in regulating vesicle fusion and neurotransmitter release, our data provide a potential mechanism underlying the synaptopathology involved in ASD.

## Limitation

The results of this study should be interpreted carefully due to several limitations. One of the primary limitations is the relatively small sample size, which is a common constraint in postmortem brain studies of ASD.^68^ While our findings provide valuable preliminary insights, they should be considered hypothesis‐generating and require further validation in larger, independent cohorts. Additionally, our analysis was limited to male brain samples, preventing an assessment of potential sex differences in DNA methylation patterns. Another important limitation is the lack of additional validation experiments, such as EM‐amplicon sequencing, to confirm the differentially methylated CpG sites identified in our analysis. Due to the limited availability of postmortem brain tissue and the small quantity of extracted DNA, conducting further validation was not feasible. Future studies with larger sample sizes and sufficient DNA quantities will be essential to confirm our findings and further explore their biological significance. Furthermore, multiple comparison correction was not performed due to the small sample size, which reduces the statistical power of the analysis. While stringent correction methods, such as Bonferroni, could help control for false‐positives, they may also lead to an overly conservative approach, potentially masking biologically meaningful differences.^41^ Given the exploratory nature of this study, we applied a more lenient significance threshold to balance the risk of type I errors with the need to detect relevant DNA methylation changes. Despite these limitations, this study provides an initial exploration of genome‐wide DNA methylation patterns in the DR region of individuals with ASD, highlighting its potential relevance. We will continue collecting more samples and conducting epigenomic and transcriptome analyses to further investigate these findings. When additional postmortem brain samples become available, we intend to perform further DNA methylation analyses using the EPIC array to expand the coverage of CpG sites. In these future studies, we will also make every effort to include female samples whenever feasible, allowing for a more comprehensive investigation of potential sex differences in DNA methylation patterns. Our findings contribute to the ongoing research on the role of serotonergic systems in ASD pathophysiology. Moving forward, increasing the sample size and incorporating additional epigenomic and transcriptomic analyses may help further clarify the relationship between DNA methylation and gene expression alterations in both brain and blood. These efforts could ultimately aid in identifying candidate biomarkers and advancing our understanding of the molecular mechanisms associated with ASD.

## Conclusions

Our findings reveal extensive DNA methylation changes in critical genomic regions, shedding light on potential mechanisms underlying ASD. Notably, we found that heightened expression of *RABGGTB*, a gene related to autophagy and synaptic function, aligns with the hypomethylation of its promoter‐associated CGIs. *RABGGTB* is a novel candidate gene absent from the ASD‐related genes database (SFARI database), emphasizing the importance of further research into its involvement in ASD and its potential as a diagnostic marker. Nevertheless, it is possible that the complex methylation systems identified in the present study, including the methylation of the promoter regions and the intragenic and intergenic regions, are involved in the pathogenesis of ASD. In future studies, it will be important to combine DNA methylation profiling with transcriptome analyses to evaluate the relationship between aberrant DNA methylation and RNA expression, including the association between DNA methylation status at alternative promoters and the expression levels of transcript variants. This study presents the first comprehensive analysis of DNA methylation in the DR nucleus, a region not previously investigated in this context. The findings contribute to a broader understanding of methylation abnormalities in ASD and may help elucidate the overall landscape of epigenetic regulation associated with the disorder.

## Disclosure statement

Kazuhiko Nakamura is an editorial board member of *Psychiatry and Clinical Neurosciences* and a co‐author of this article. To minimize bias, they were excluded from all editorial decision‐making related to the acceptance of this article for publication.

## Author contributions

Keiko Iwata: study concept and design; acquisition of data; analysis and interpretation of data; writing of the manuscript for content. Kazuhiko Nakabayashi: acquisition of data; analysis and interpretation of data; writing of the manuscript for content. Keisuke Ishiwata: acquisition of data; analysis and interpretation of data. Kazuhiko Nakamura: sample collection; writing of the manuscript for content. Yosuke Kameno: acquisition of data; analysis and interpretation of data. Kenichiro Hata: study concept and design; acquisition of data; analysis and interpretation of data; writing of the manuscript for content. Hideo Matsuzaki: study concept and design; acquisition of data; analysis and interpretation of data; writing of the manuscript for content.

## Supporting information


**Figure S1.** Integrative genomics viewer–based view of the c. I‐Mb genomic regions detected around intergenic differentially methylated regions (DMRs). Intergenic DMR located just before HTR2C. Average methylation values that were significantly (*P* < 0.05) increased or decreased in patients with autism spectrum disorder (ASD) compared with controls are shown (blue bars). The average methylation values are expressed as *β*‐values (0–1). The Delta *β*‐value is colored in pink (increased).

**Figure S2.** DNA methylation rates at cg08702915 (chrl: 76,253,688 (hgl 9)) located in the intron 2 of *RABGGTB*. Methylated (red) and unmethylated (blue) rates.

**Figure S3.** Expression of the *COLECII*, *DCAKD*, *RABGGTB*, and *ZBTB40* genes in the raphe region of postmortem brains. Comparison of the gene levels in the raphe region of postmortem brains from controls and patients with autism spectrum disorder (ASD). Data are presented as mean ± SEM. *n* = 7 male controls and *n* = 5 male patients with ASD. Mann–Whitney *U* test was used to evaluate the gene expression levels.

**Figure S4.** Correlations between expression and methylation of the *COLECII*, *DCAKD*, *RABGGTB*, and *ZBTB40* genes in the raphe region of postmortem brains. The analysis examined the relationship between gene expression levels and their corresponding methylation levels (AVG β‐values) obtained from the methylation array at each site in the raphe region of postmortem brains from control individuals and patients with autism spectrum disorder (ASD). The cohort included *n* = 7 male controls and *n* = 5 male patients with ASD. Spearman rank correlation coefficient was employed to assess the relationship between methylation levels and gene expression.

**Table S1.** Information on postmortem brain tissues.

**Table S2.** Demographic data associated with raphe brain tissue samples using methylation analyses.

**Table S3.** Demographic data associated with raphe brain tissue samples for the gene expression analysis.

## Data Availability

DNA methylation data have been deposited in GEO (accession number GSE242427).
