# Supplementary figures and images for: Genome‐wide DNA methylation profiles in the raphe nuclei of patients with autism spectrum disorder
Source: Psychiatry Clin Neurosci. 2025 Apr 24;79(7):415–24. doi: 10.1111/pcn.13830 (PMC12232043; doi:10.1111/pcn.13830)

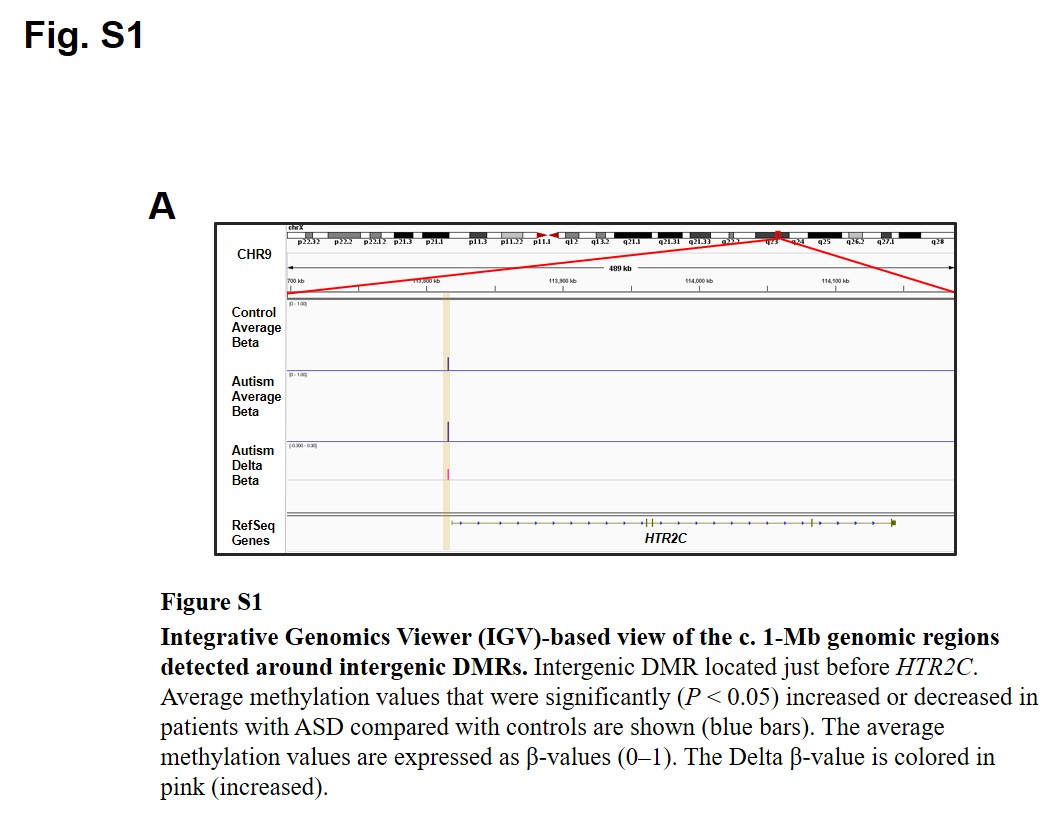

Supplement: Supplementary file 1 — Figure S1. Integrative genomics viewer–based view of the c. I‐Mb genomic regions detected around intergenic differentially methylated regions (DMRs). Intergenic DMR located just before HTR2C. Average methylation values that were significantly (P < 0.05) increased or decreased in patients with autism spectrum disorder (ASD) compared with controls are shown (blue bars). The average methylation values are expressed as β‐values (0–1). The Delta β‐value is colored in pink (increased). [file PCN-79-415-s001.jpg]

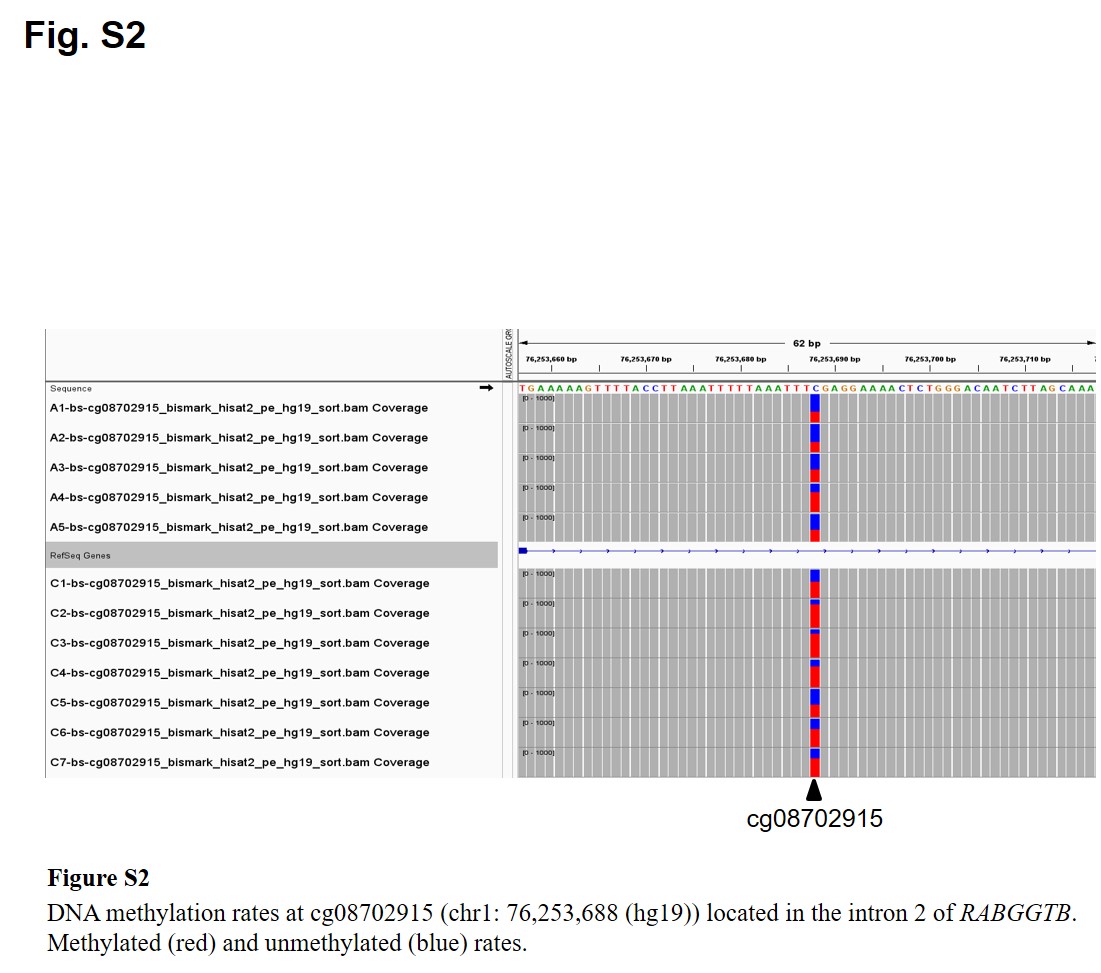

Supplement: Supplementary file 2 — Figure S2. DNA methylation rates at cg08702915 (chrl: 76,253,688 (hgl 9)) located in the intron 2 of RABGGTB. Methylated (red) and unmethylated (blue) rates. [file PCN-79-415-s002.jpg]

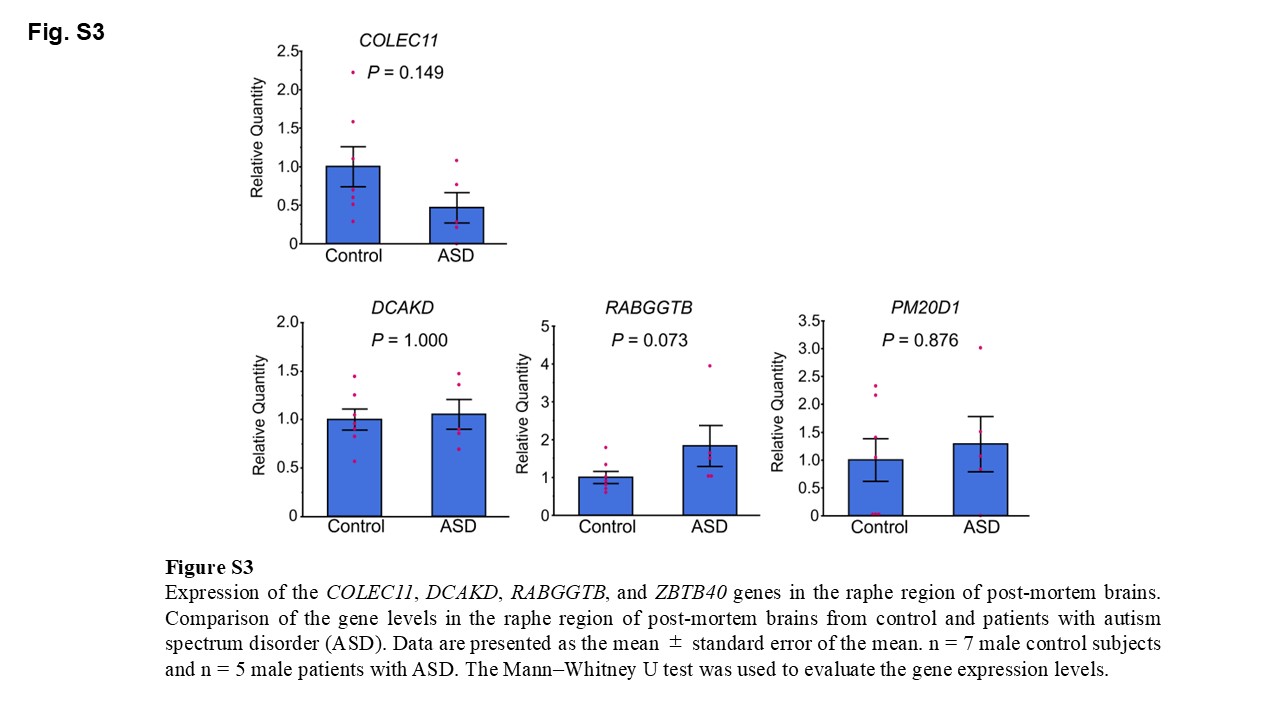

Supplement: Supplementary file 3 — Figure S3. Expression of the COLECII, DCAKD, RABGGTB, and ZBTB40 genes in the raphe region of postmortem brains. Comparison of the gene levels in the raphe region of postmortem brains from controls and patients with autism spectrum disorder (ASD). Data are presented as mean ± SEM. n = 7 male controls and n = 5 male patients with ASD. Mann–Whitney U test was used to evaluate the gene expression levels. [file PCN-79-415-s005.jpg]

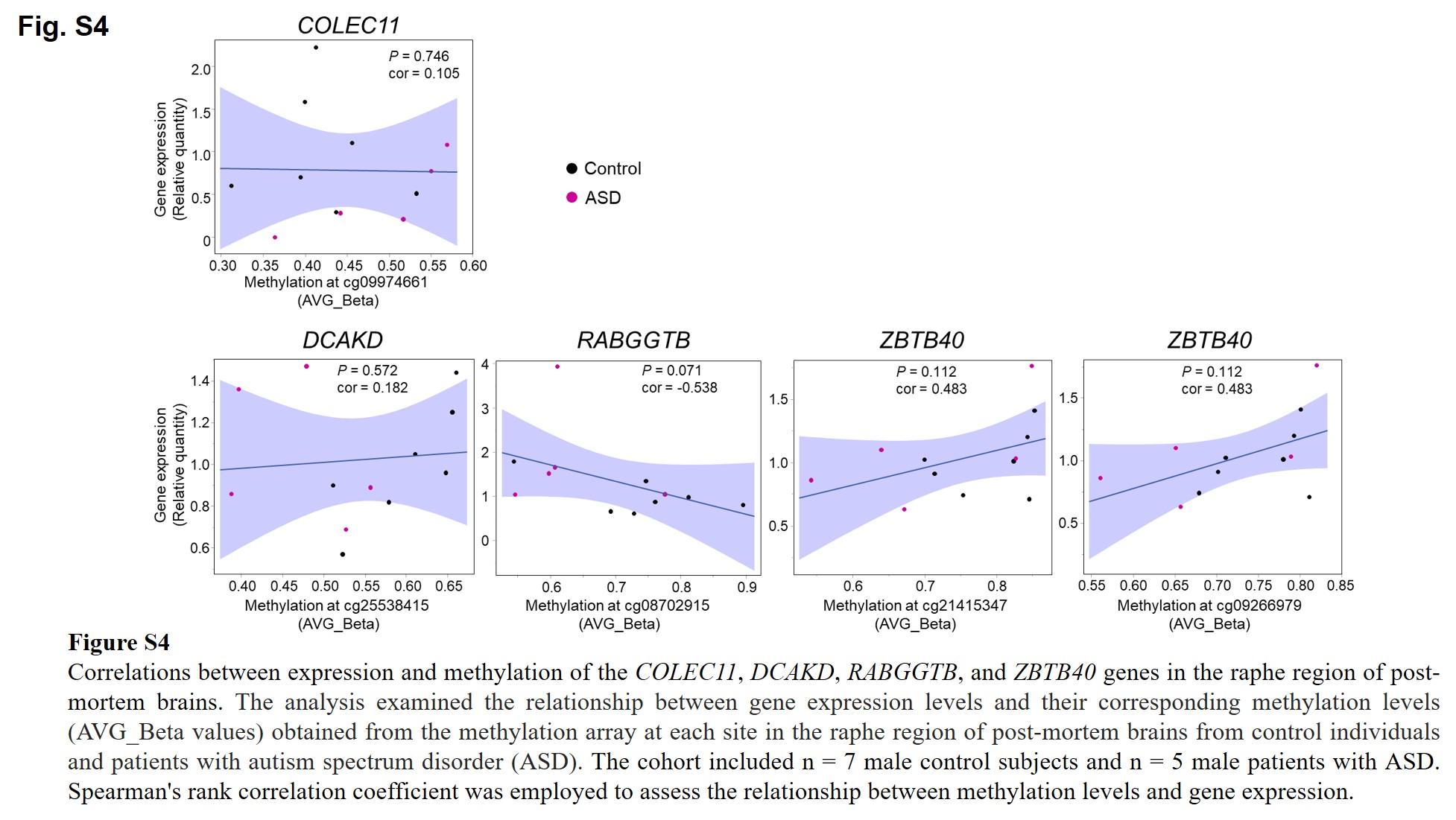

Supplement: Supplementary file 4 — Figure S4. Correlations between expression and methylation of the COLECII, DCAKD, RABGGTB, and ZBTB40 genes in the raphe region of postmortem brains. The analysis examined the relationship between gene expression levels and their corresponding methylation levels (AVG β‐values) obtained from the methylation array at each site in the raphe region of postmortem brains from control individuals and patients with autism spectrum disorder (ASD). The cohort included n = 7 male controls and n = 5 male patients with ASD. Spearman rank correlation coefficient was employed to assess the relationship between methylation levels and gene expression. [file PCN-79-415-s004.jpg]
